# Integrating automated liquid handling in the separation workflow of extracellular vesicles enhances specificity and reproducibility

**DOI:** 10.1186/s12951-023-01917-z

**Published:** 2023-05-19

**Authors:** Sofie Van Dorpe, Lien Lippens, Robin Boiy, Cláudio Pinheiro, Glenn Vergauwen, Pekka Rappu, Ilkka Miinalainen, Philippe Tummers, Hannelore Denys, Olivier De Wever, An Hendrix

**Affiliations:** 1 Laboratory of Experimental Cancer Research, Department of Human Structure and Repair, Ghent University, Ghent, Belgium; 2 Cancer Research Institute Ghent, Ghent, Belgium; 3 Department of Gynecology, Ghent University Hospital, Ghent, Belgium; 4 Department of Life Technologies, University of Turku, Turku, Finland; 5 Biocenter Oulu, University of Oulu, Oulu, Finland; 6 Department of Medical Oncology, Ghent University Hospital, Ghent, Belgium

**Keywords:** Extracellular vesicles, Separation, Density gradient centrifugation, Blood, Urine, Automation, Proteomics

## Abstract

**Background:**

Extracellular vesicles (EV) are extensively studied in human body fluids as potential biomarkers for numerous diseases. Major impediments of EV-based biomarker discovery include the specificity and reproducibility of EV sample preparation as well as intensive manual labor. We present an automated liquid handling workstation for the density-based separation of EV from human body fluids and compare its performance to manual handling by (in)experienced researchers.

**Results:**

Automated versus manual density-based separation of trackable recombinant extracellular vesicles (rEV) spiked in PBS significantly reduces variability in rEV recovery as quantified by fluorescent nanoparticle tracking analysis and ELISA. To validate automated density-based EV separation from complex body fluids, including blood plasma and urine, we assess reproducibility, recovery, and specificity by mass spectrometry-based proteomics and transmission electron microscopy. Method reproducibility is the highest in the automated procedure independent of the matrix used. While retaining (in urine) or enhancing (in plasma) EV recovery compared to manual liquid handling, automation significantly reduces the presence of body fluid specific abundant proteins in EV preparations, including apolipoproteins in plasma and Tamm-Horsfall protein in urine.

**Conclusions:**

In conclusion, automated liquid handling ensures cost-effective EV separation from human body fluids with high reproducibility, specificity, and reduced hands-on time with the potential to enable larger-scale biomarker studies.

**Graphical Abstract:**

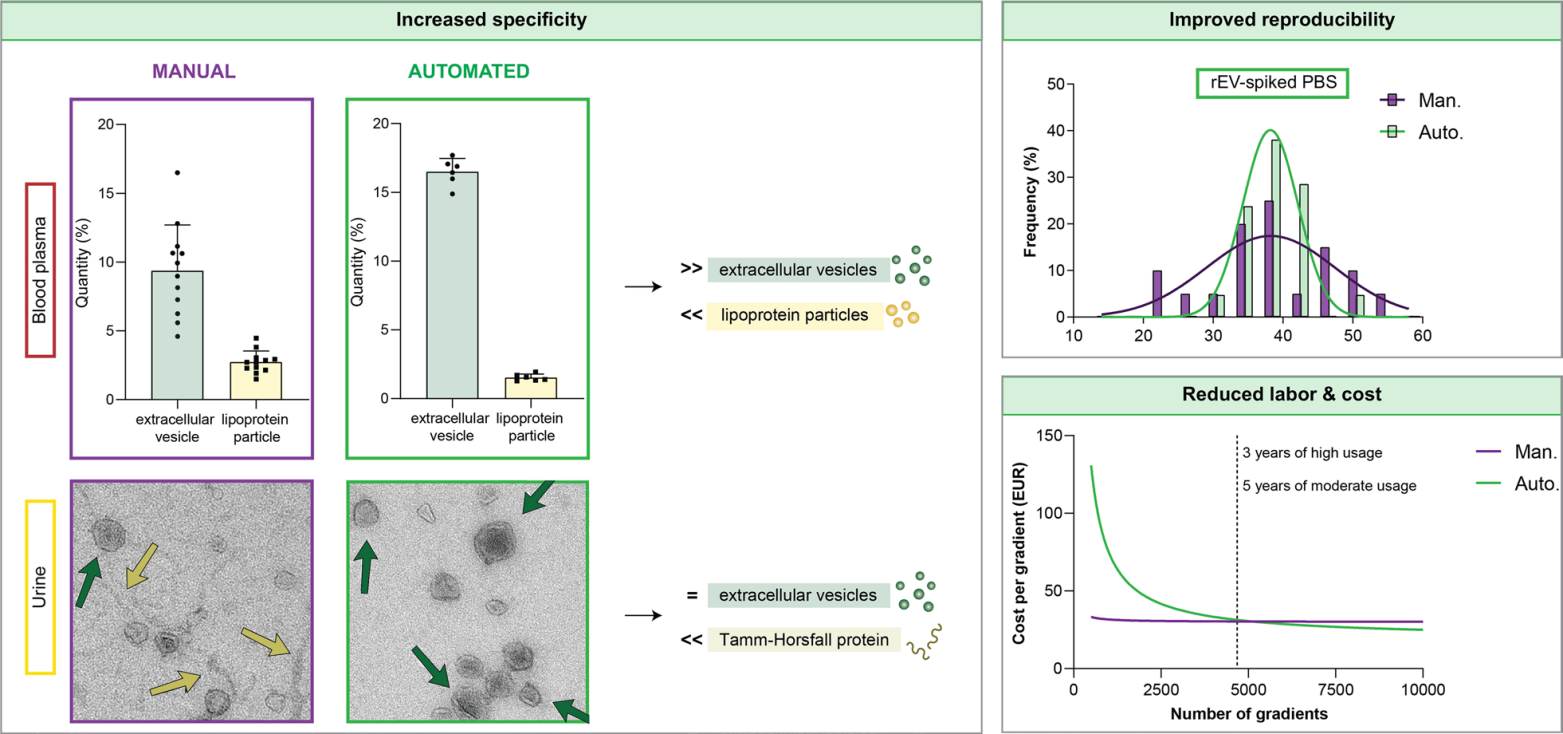

**Supplementary Information:**

The online version contains supplementary material available at 10.1186/s12951-023-01917-z.

## Background

Extracellular vesicles (EV) in body fluids are increasingly explored as biomarkers for diagnosis, prognosis, and therapy surveillance in various diseases including cancer [1]. These nanosized membrane vesicles transport proteins, nucleic acids, and lipids among cells and their molecular cargo corresponds to the current state of the releasing cells [2]. EV are released in easily accessible body fluids, including blood and urine, which makes non-invasive and repetitive sampling possible [3].

EV-based biomarker discovery requires highly specific, reproducible, and standardized EV separation [3–6]. EV have been separated from body fluids using a wide diversity of methods including differential ultracentrifugation, size-exclusion chromatography (SEC), immunoprecipitation and density gradient centrifugation [7–10]. Automation has recently been introduced in multiple EV separation workflows. Examples include automated fraction collection, measuring fluid volume by weight [11] and fully integrated microfluidic systems combining immunoaffinity, filtration, centrifugation, and/or asymmetrical flow field-flow fractionation [12–16].

EV separation methods co-isolate to various degrees EV with other biological components [9, 17]. Density gradient centrifugation outperforms other methods (including SEC and differential ultracentrifugation) in increasing specificity of EV preparations from cell culture supernatant, blood plasma, and urine [8, 9, 18, 19]. Buoyant density separates EV from lipoprotein particles and protein aggregates in blood; and from Tamm-Horsfall protein (THP) complexes and soluble proteins in urine [9, 10, 20]. In general, a combination of techniques is necessary to first concentrate EV and then increase specificity by separating EV from non-EV components [17]. To separate EV with high specificity from human body fluids and enable biomarker discovery, we previously reported the orthogonal implementation of size- (SEC and ultrafiltration) and density-based methods (density gradient centrifugation) [10, 21]. Similar orthogonal approaches involving density-based methods for the study of EV in blood plasma and urine have been described by other research groups [22–27].

To prepare a discontinuous density gradient, decreasingly dense solutions are layered on top of each other ensuring sharp interfaces between densities [28]. The manual procedure requires hands-on skills since the layering technique is tedious and time-consuming, and is often not reproducible among operators because of insufficient training and lack of a steady hand and patience [28]. After centrifugation, gradients are typically fractionated by manually pipetting from the meniscus. As with gradient preparation, this procedure is prone to error due to the challenge to collect fractions of equal volume. Expert training is needed to keep the tip of the pipette at the meniscus without occasionally aspirating some air or liquid from below the meniscus. Overcoming human variables, robotic automation may enable consistent preparation of discontinuous density gradients with sharp interfaces between layers and precise collection of equal volumes exactly at the liquid surface by liquid-level sensing. The use of automated density gradient preparation and fraction collection has been reported in scientific papers and technical notes but has not been compared to the manual procedure [8, 10, 29, 30].

**Fig. 1:**
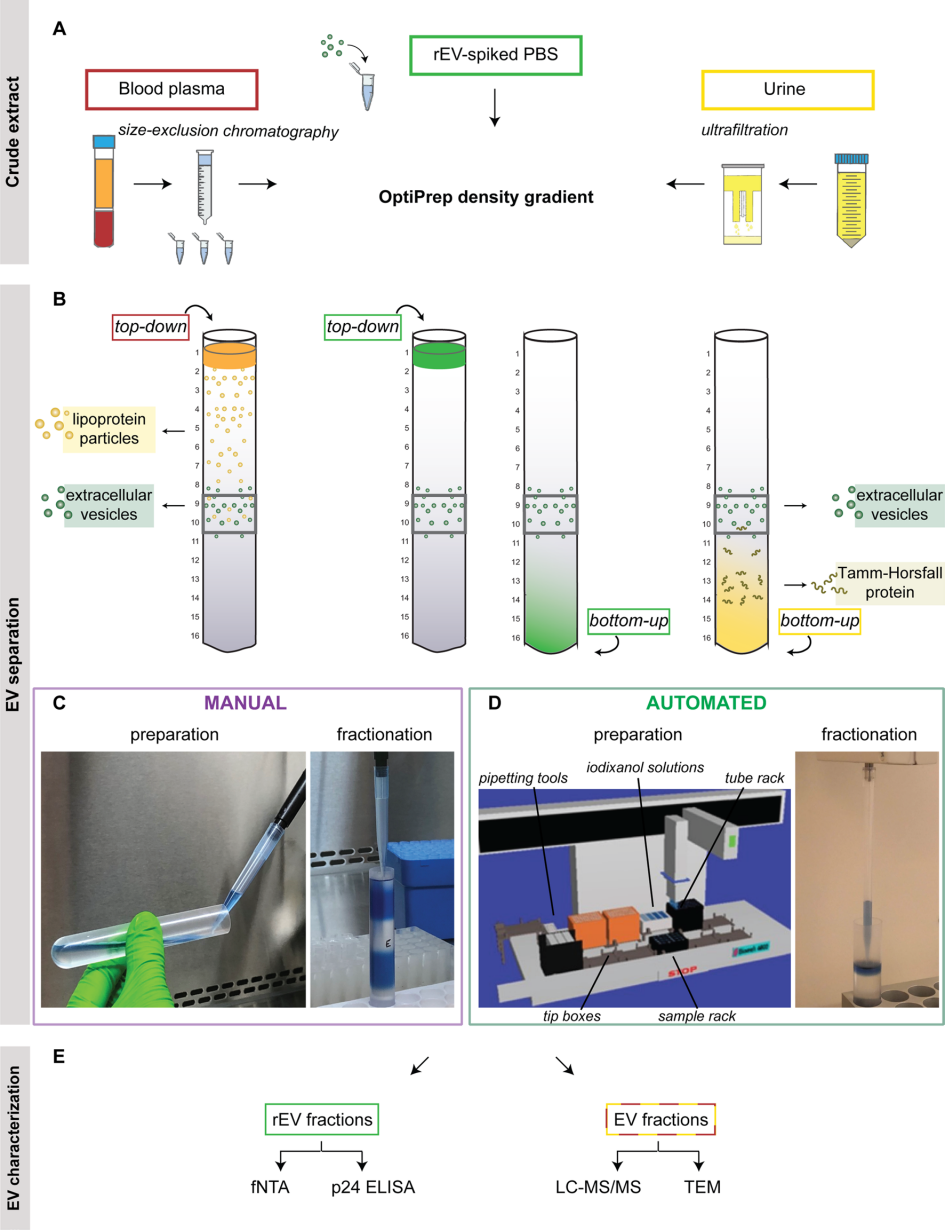
Illustrative overview of the EV separation protocol.** A** Separation of EV from [1] PBS spiked with recombinant EV (rEV), and from body fluids [2] blood plasma and [3] urine by orthogonal biophysical methods. **B** OptiPrep density gradient centrifugation of rEV-spiked PBS (both top-down (TD) and bottom-up (BU) approach), crude blood plasma extracts (TD approach) and concentrated urine (BU approach). **C** Manual preparation of a density gradient with the addition of 100 µL 0.4% (wt/vol) trypan blue solution to the 20% and 5% (wt/vol) iodixanol solutions. The centrifuge tube is tilted to 70° and the 20% iodixanol solution is carefully, dropwise transferred to the surface of the liquid. Manual fraction collection in which the tube is being held upright and fractions are carefully collected by slowly pipetting 1 mL from the central bottom of the concave meniscus at the liquid surface. **D** Visualization of the Biomek 4000 automated workstation setup for density gradient preparation. Automated fraction collection with liquid-level sensing enabling precise collection of the upper 1 mL volume on the liquid surface. **E** Characterization of rEV-enriched fractions by fluorescent nanoparticle tracking analysis (fNTA) and p24 ELISA, and of EV preparations obtained from blood plasma and urine by LC-MS/MS and transmission electron microscopy (TEM)

In this study, we evaluated automated liquid handling for the density-based separation of EV from human body fluids and compared its performance to manual handling by (in)experienced operators (Fig. 1). Variability in density-based EV separation using manual versus automated liquid handling was first evaluated by making use of trackable recombinant extracellular vesicles (rEV) [29]. Next, we compared variability, EV recovery, and specificity in EV preparations obtained from blood plasma and urine by mass spectrometry-based proteomics and transmission electron microscopy. Other performance parameters we explored include hands-on time and cost.

## Results

### Automatic liquid handling reduces interface mixing during density gradient preparation

**Fig. 2:**
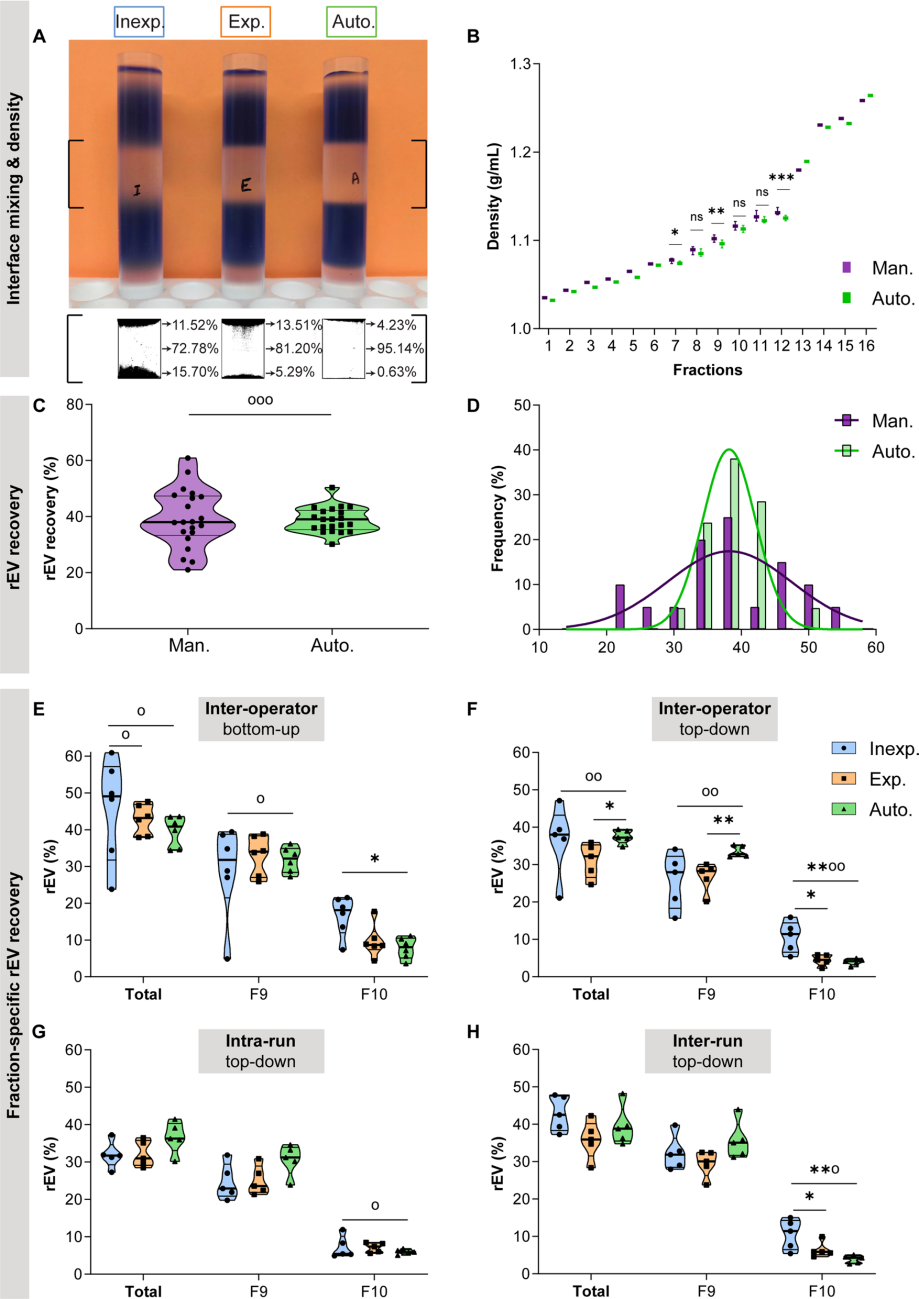
Reproducibility of density-based rEV separation by (in)experienced operators versus automated liquid handling. **A** Visualization of the accuracy in representative colored test gradients prepared by an inexperienced (inexp.), experienced (exp.), and automated operator (auto.) and the spilling of the 5% and 20% iodixanol solution in the 10% layer. **B** Density of the collected fractions obtained by manual (man.) versus automated density gradient centrifugation (fractions 7–12: n = 19,) as determined by 340 nm absorbance. **C**,** D** Recombinant EV (rEV) recovery (as quantified by the mean of fNTA and anti-p24 ELISA measurements) after bottom-up (BU) (n = 11) and top-down (TD) (n = 10) density gradient centrifugation by manual and automated liquid handling. **E** Inter-operator variability total and fraction-specific rEV recovery in after bottom-up density-based separation by (in)experienced operators versus automated liquid handling (n = 6). Total and fraction-specific rEV recovery after top-down density-based separation by (in)experienced operators versus automated liquid handling (n = 5) to compare **F** inter-operator, **G** intra-run and **H** inter-run variability. ***P < 0.001, **P < 0.01, *P < 0.05 (Mann–Whitney U test). °°P < 0.01, °P < 0.05 (F-test of equality of variances). Data in C, E, F, G, H are depicted as truncated violin plots. Data in D are depicted as histograms and accompanying gaussian curves. Source data are provided as Additional file 7: Table S1

Density gradient preparation and fraction collection was performed manually (man.), by inexperienced (inexp.) or experienced (exp.) operators, or automatic (auto.). A representative colored test gradient prepared by an inexperienced operator showed a blurry interface between the different density layers with an interfacial area of 27.22% of the total area. An experienced operator reduced this interfacial mixing to 18.80% of the total area, while automated liquid handling, sharply defined this interfacial area to only 4.86% of the total area (Fig. 2A). Density measurements consistently identified lower density values in the collected fractions 7–12 obtained by automated versus manual density gradient centrifugation (n = 18 per group) (Fig. 2B). Density fractions 9 and 10, commonly analyzed as EV-enriched density fractions [8, 9, 18, 27, 31–35], had a mean density of respectively 1.096 g/mL and 1.113 g/mL by automated liquid handler preparation compared to 1.102 g/mL and 1.116 g/mL by manual preparation (Mann-Whitney U test, respectively p = 0.009 and p = 0.15).

### Automation and hands-on training significantly reduce variability in bottom-up and top-down density-based EV separation

We compared variability in the separation of rEV spiked in PBS (1.85 × 10^10^ rEV per gradient as quantified by fNTA) by manual and automated liquid handling using BU or TD density gradient centrifugation (Fig. 1A, B). Hereto, we quantified two characteristics unique to rEV: fluorescence intensity (measured by fNTA) and the presence of the HIV gag protein (measured by anti-p24 ELISA) [29]. As previously described [29], quantification of rEV by fNTA and ELISA revealed a positive linear correlation (Pearson’s r = 0.85, R² = 0.73, p < 0.0001) (Additional file 1: Fig. S1B) and rEV showed a size between 50 and 250 nm (Additional file 1: Fig. S1D, E). rEV contain EV-associated proteins, including CD9, ALIX, and flotillin-1 as assessed by western blot analysis (Additional file 1: Fig. 1C) and previously reported [29]. rEV recovery after density-based separation was calculated by the number of rEV in density fraction 9 and 10 (as quantified by the mean of fNTA and anti-p24 ELISA measurements), divided by the total number of spiked rEV. Technical variability in rEV recovery was assessed by calculating the coefficient of variation (CV). Overall, automated liquid handling significantly reduced variability in total rEV recovery after BU and TD density-based separation (CV_auto_ 11.58% vs. CV_man_ 26.44%, F-test, p = 0.0004) (Fig. 2C, D).

Next, we evaluated inter-operator (different operators, same time point), intra-run (same operator, same time point), and inter-run (same operator, different time points) variability in rEV recovery.

For BU density-based separation by six different operators, inter-operator variability in total rEV recovery (= sum of fraction 9 and 10) was significantly higher for inexperienced operators compared to experienced operators (F-test, p = 0.018) and automated liquid handling (p = 0.020) (Fig. 2E). For TD density-based separation by five different operators, inter-operator variability in total rEV recovery was also significantly higher for inexperienced operators compared to automation (p = 0.008) (Fig. 2F). The inter-operator CV_auto_ for total rEV recovery was 10.63% (BU) and 4.94% (TD), CV_exp_ was 9.60% (BU) and 14.85% (TD), and CV_inexp_ was 30.53% (BU) and 26.06% (TD). The inter-operator variability was significantly lower for fraction-specific rEV recovery by automated versus inexperienced manual liquid handling in BU fraction 9 (F-test, p = 0.012), and in TD fraction 9 (p = 0.009) and 10 (p = 0.009) (Fig. 2E, F). Automated liquid handling recovered significantly more total rEV and rEV in TD fraction 9 compared to experienced manual liquid handling (Mann-Whitney U test, respectively p = 0.016 and p = 0.008). In contrast, significantly fewer rEV were recovered by automated versus inexperienced manual liquid handling in BU fraction 10 (p = 0.015) and TD fraction 10 (p = 0.008) and by experienced versus inexperienced manual liquid handling in TD fraction 10 (p = 0.032).

To assess intra-run variability, five TD density gradients were prepared and fractionated at the same time point by an (in)experienced operator or by the automated workstation. No significant differences in intra-run variability were observed in total rEV recovery (intra-run CV_auto_ 11.60%, CV_exp_ 10.77%, and CV_inexp_ 11.21%). Fraction-specific rEV recovery by automated liquid handling showed significantly lower intra-run variability versus inexperienced manual liquid handling in fraction 10 (F-test, 0.013) (Fig. 2G).

To evaluate inter-run variability, the same (in)experienced operator and the automated liquid handler performed TD density gradient centrifugation on five different time points. No significant differences in inter-run variability were observed in total rEV recovery (CV_auto_ 13.22%, CV_exp_ 14.17%, and CV_inexp_ 10.91%). Inter-run variability was significantly lower for fraction-specific rEV recovery by automated versus inexperienced manual liquid handling in fraction 10 (F-test, p = 0.022) (Fig. 2H). While retaining equal total rEV recovery, significantly fewer rEV were recovered by automated versus in- and experienced manual liquid handling in fraction 10 (Mann-Whitney U test, p_inexp_ = 0.008, p_exp_ = 0.016).

In conclusion, inexperienced operators showed the largest inter-operator variability for total rEV recovery (mean CV 28.29%). For automated liquid handling the intra-run and inter-operator variability in total rEV recovery were lowest (mean CV 9.06%).

### Validation of reproducible automated density-based EV separation from human body fluids

Since automated liquid handling significantly reduced inter-operator variability in the separation of rEV spiked in PBS, we decided to validate this observation using human body fluids. Methodological replicates (n = 6 per group) derived from a pool of crude blood plasma extracts collected from breast cancer patients and processed by SEC were subjected to TD density gradient centrifugation and methodological replicates (n = 6 per group) derived from a pool of concentrated cell-free urine collected from healthy volunteers were subjected to BU density gradient centrifugation (Fig. 1A, B). EV-enriched density fractions 9 and 10 were each analyzed by mass-spectrometry based proteomics (LC-MS/MS). Fraction 9 and 10 revealed respectively 1009 and 517 proteins for blood plasma-derived EV, and 2030 and 2626 proteins for urine-derived EV (Additional file 1: Fig. S2A, Additional file 8: Table S2). A total of 258 common proteins were identified in all EV preparations, including EV-associated proteins CD9, CD63, ALIX, MSN, and ANXA1. 243 and 2216 proteins were uniquely identified in respectively blood plasma- and urine-derived EV.

**Fig. 3:**
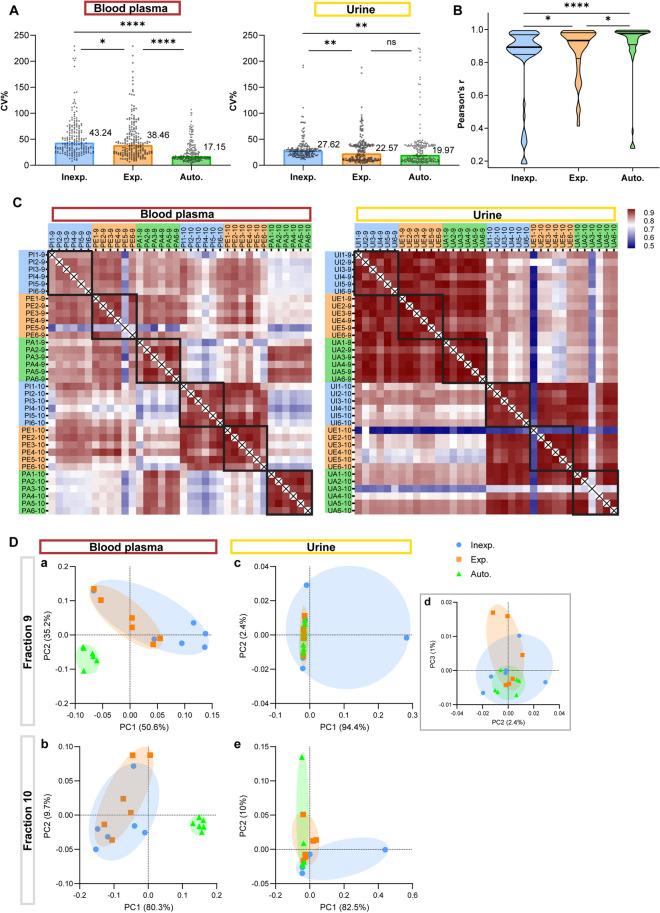
Technical analysis of density-based EV separation of blood plasma and urine by mass spectrometry-based proteomic analysis (LC-MS/MS). LC-MS/MS data from EV-enriched density fractions 9 and 10 obtained from blood plasma and urine after density-based separation by inexperienced (inexp.), experienced (exp.) operators, and automated liquid handling (auto.) (n = 6) are compared by **A** coefficients of variation (CV) for protein group quantification, **B**, **C** correlation analysis, and **D** principal component analysis. Correlation is represented as Pearson’s r coefficient in a heatmap and violin plots. **** P < 0.0001, **P < 0.01, *P < 0.05 (Mann–Whitney U test). Source data are provided as Additional file 8: Table S2

Method reproducibility was highest in the automated procedure. The CV_auto_ for protein group quantification was 17.2% (plasma) and 20.0% (urine), CV_exp_ was 38.5% (plasma) and 22.6% (urine), and CV_inexp_ was 43.2% (plasma) and 27.6% (urine) (Fig. 3A). Anosim analysis based on LFQ intensities demonstrated high similarity between samples with equal density fraction, body fluid type, and operator type (R = 0.8323, p = 0.0001) (Additional file 1:  Fig. S2B). Correlation analysis confirmed high similarity between these samples with median Pearson’s r_auto_ of 0.98, r_exp_ of 0.93, and r_inexp_ of 0.89 (Fig. 3B, C). A significant higher correlation was detected between automated EV preparations compared to (in) experienced manual EV preparations (Mann-Whitney U test, p_auto−inexp_ < 0.0001, p_auto−exp_ = 0.025) (Fig. 3B). Notably, the correlation between manual EV preparations from experienced operators was significantly higher compared to preparations from inexperienced ones (p_exp−inexp_ = 0.045) (Fig. 3B). Likewise, in the principal component analysis (PCA) plots based on LFQ intensities we observed a higher similarity between the methodological replicates within the automated clusters (Fig. 3D). Since PC1 in urine fraction 9 was mostly determined by THP in one of the inexperienced replicates, we included PC3 which also confirmed wider spreading in manual versus automated samples.

Interestingly, PCA of the blood plasma density fractions showed differential clustering of the manual and automated EV preparations, whereas clusters overlapped in urine (Fig. 3D). This observation is confirmed by the correlation analysis plot of blood plasma which demonstrated a poor correlation between manual and automated EV preparations within the same fraction (Fig. 3C). Unsupervised hierarchical cluster analysis showed that urine EV preparations group first by density fraction and then by operator, whereas blood plasma EV preparations group first by operator and then by density fraction (Additional file 1: Fig. S2C). In conclusion, improved inter-operator reproducibility by automated density-based EV separation, observed for rEV spiked PBS, was confirmed in complex human body fluids.

### Automated liquid handling enhanced the specificity of EV preparations from human body fluids

**Fig. 4:**
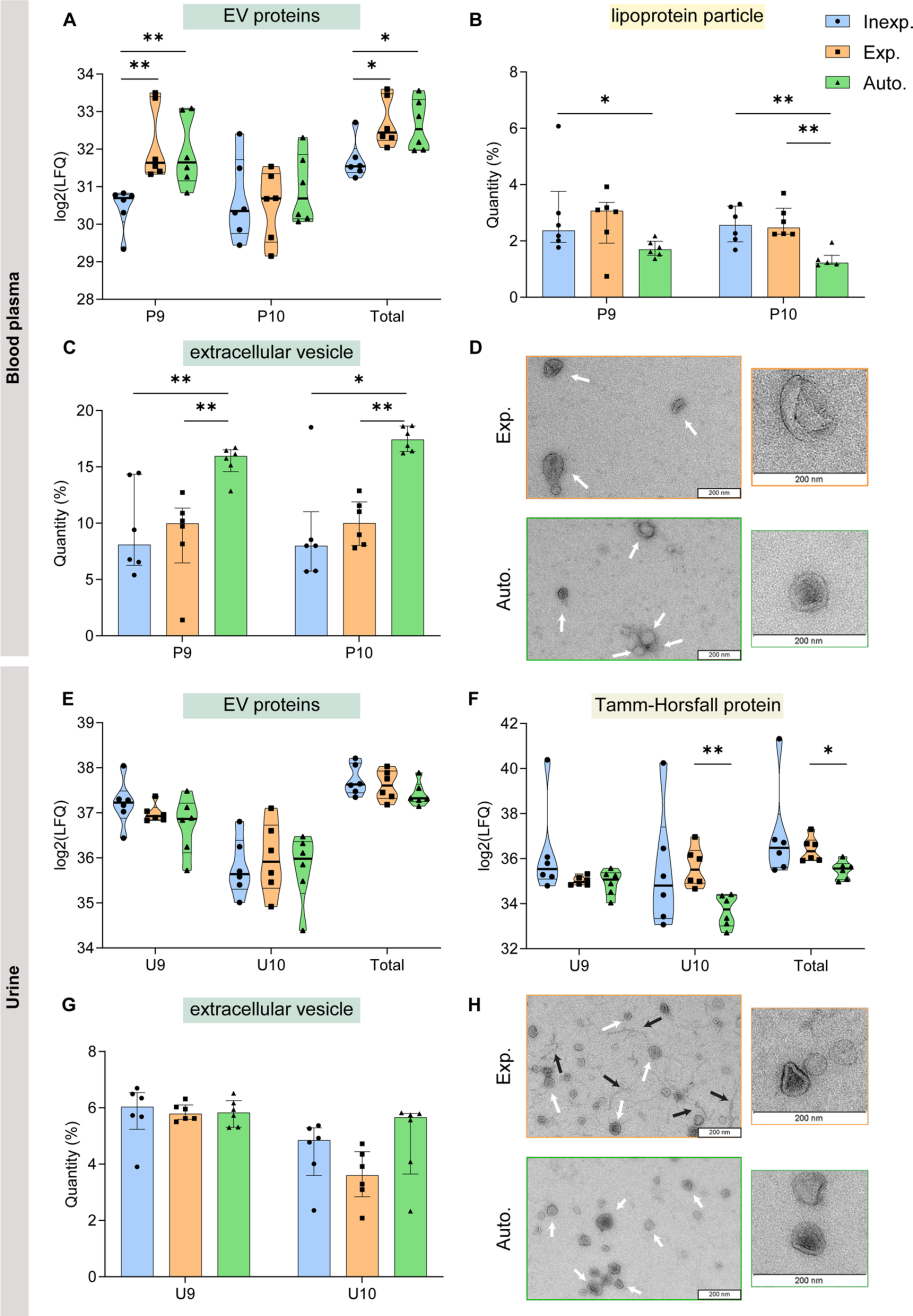
Density-based EV separation from blood plasma and urine by (in)experienced operators versus automated liquid handling. **A** Log2 of LFQ intensities of EV-associated proteins (CD9, CD63, ALIX (PDCD6IP), ANXA1, MSN, GAPDH, GNAI2, HSPA8) in density fractions 9 and 10 obtained from blood plasma after density-based separation by (in)experienced operators (inexp., exp.) and automated liquid handling (auto.). LFQ intensity-based enrichment analysis of density fractions 9 and 10 obtained from blood plasma comparing enrichment in **(B)** ‘lipoprotein particle’ and **C** ‘extracellular vesicle’ GOCC terms. **D** Transmission electron microscopy of EV-enriched density fractions obtained from blood plasma by an experienced operator and automated liquid handling (scale bar: 200 nm). Log2 of LFQ intensities of **(E)** EV-associated proteins and **F** Tamm-Horsfall protein in density fractions 9 and 10 obtained from urine after density-based separation by (in)experienced operators and automated liquid handling. **G** LFQ intensity-based enrichment analysis of density fractions 9 and 10 obtained from urine comparing enrichment in ‘extracellular vesicle’ GOCC term. **H** Transmission electron microscopy of EV-enriched density fractions obtained from urine by an experienced operator and automated liquid handling. White arrows indicate EV and black arrows indicate THP polymers. **P < 0.01, *P < 0.05 (Mann–Whitney U test). Source data are provided as Additional file 8:  Table S2

The log2 of summed LFQ intensities of EV-associated proteins (CD9, CD63, ALIX (PDCD6IP), ANXA1, MSN, GAPDH, GNAI2, HSPA8) were significantly higher in EV preparations by experienced operators and automated liquid handling versus inexperienced operators (Mann-Whitney U test, P9, p_auto−inexp_ = 0.002, p_exp−inexp_ = 0.002; total, p_auto−inexp_ = 0.015, p_exp−inexp_ = 0.026) (Fig. 4A). Indeed, quantitative enrichment analysis based on the number of input proteins and LFQ protein abundance demonstrated blood plasma fractions 9 and 10 were significantly more enriched in the Gene Ontology Cellular Component (GOCC) term ‘extracellular vesicle’ (Mann-Whitney U test, P9, p_auto−inexp_= 0.009, p_auto−exp_ = 0.002; P10, p_auto−inexp_ = 0.026, p_auto−exp_ = 0.002) after automated compared to (in)experienced manual liquid handling (Fig. 4C). Furthermore, blood plasma fractions 9 and 10 were significantly less enriched in GOCC term ‘lipoprotein particle’ after automated compared to manual liquid handling (Mann-Whitney U test, P9, p_auto−inexp_ = 0.026; P10, p_auto−inexp_ = 0.004, p_auto−exp_ = 0.002) (Fig. 4B).

In urine, the log2 of summed LFQ intensities of EV-associated proteins (Fig. 4E) and the enrichment of the term ‘extracellular vesicle’ (Fig. 4G) were similar in EV preparations by (in)experienced operators versus automated liquid handling. Interestingly, automation resulted in a significant decrease in THP compared to experienced liquid handling in urinary fraction 10 (Mann-Whitney U test, p_auto−exp_ = 0.002) and in total (p_auto−exp_ = 0.015) (Fig. 4F).

TEM of EV-preparations obtained from blood plasma (Fig. 4D) and urine (Fig. 4H) identified vesicular structures with a size between 30 and 150 nm characteristic of EV. In urine, TEM confirmed higher contamination with polymeric networks of THP by manual compared to automated liquid handling (Fig. 4H).

### Investment in automation minimized hands-on time and reduced costs

The duration of manual density gradient preparation and fractionation is 15–25 and 10–15 min per gradient, respectively. The duration depends on the experience and accuracy of the operator (Additional file 1: Fig. S3A). Automated density gradient preparation and fractionation only requires a few minutes of manual labor. The liquid handler needs 25 min to prepare one to four gradients, and 50 min for five to eight gradients. The automated fraction collection takes 8 min per gradient.

The yearly cost of density gradient preparation and fractionation depends on the initial set up cost (equipment and training) and operating cost per year (maintenance, labor, and consumables specific to the technique) (Additional file 1: Fig. S3B, Additional file 9: Table S3). The operating costs were based on moderate (18 gradients per week) or high performance of density gradient centrifugation (the in-house average, i.e. 30 gradients per week). In case of automation, the set-up cost is expensive due to the investment in the liquid handler. However, investment in training is over 700 EUR per operator in case of manual liquid handling compared to 20 EUR in case of automation. In-house, the manual operating cost of density-based EV separation per year is 1.5-fold higher compared to the automated operating cost due to its time-consuming nature and associated high labor cost.

The main disincentive to automation is the cost of initial investing in the hardware. However, automation could dramatically reduce costs over time. Expensive set-up costs of the liquid handler can be recovered within 3 years of high or 5 years of moderate usage (Additional file 1: Fig. S3C). More robust research requires fewer replicates and comprises less risk of technical mistakes, which reduces laboratory costs even more.

## Discussion

Reproducible EV separation with high specificity is crucial for both basic research and translational applications of EV [4]. Despite substantial progress, challenges in standardized EV research remain considerable [4, 6, 36]. To tackle this, we introduced an automated liquid handling workstation for the density-based separation of EV from human body fluids and compared its performance to manual handling by (in)experienced operators. Performance parameters we have addressed include variability, EV recovery, and specificity.

We first spiked pre-defined numbers of rEV [29] in PBS to assess the impact of the operator (i.e. (in)experienced or automated) on recovery. For both TD and BU density gradients, automated liquid handling outperformed (in)experienced operators in recovering EV with the highest reproducibility, despite similar recovery. Evaluation of intra-run, inter-run, and inter-operator variability revealed that automated liquid handling resulted in lowest variability in total rEV recovery, but also that the distribution among the individual automated fractions was more consistent. We further validated increased reproducibility by automated liquid handling in endogenous EV preparations from complex body fluids. Correlation, anosim, and principal component analyses demonstrated that automated liquid handling prepares highly reproducible proteomics from blood plasma- and urine-derived EV. By comparing inexperienced and experienced operators, our data support the necessity of hands-on training in density gradient preparation and fraction collection to obtain consistent results. In case an automated liquid handler is not available, we recommend intensive training (Additional file 1: Box S1) and evaluation (Additional file 1: Box S2) of the technique.

Interestingly, automated liquid handling did not only increase reproducibility, but also specificity of the EV preparations. Although contamination is still detectable, it is significantly diminished by automated liquid handling. We believe accurate, dropwise sample loading in top-down gradients by automated liquid handling minimizes the risk of flushing non-EV components through the EV-enriched fractions resulting in reduced apolipoprotein contamination and enhances EV recovery in blood plasma-derived EV preparations. In urine, liquid level sensing in automated fraction collection could explain less liquid aspiration from the underlying THP-rich fractions resulting in reduced THP contamination in EV preparations. This hypothesis is supported by higher densities measured in EV-enriched fractions by manual compared to automatic prepared gradients.

In addition to increasing specificity and reproducibility, automated liquid handlers can greatly increase the throughput of a laboratory, and free up researchers for scientific creativity instead of monotonous tasks.

Different separation methods differentially enrich for EV subpopulations [6, 17]. By combining size- and density-based methods, we aim to minimize contamination while enhancing EV recovery. However, a limitation of this approach is the possible exclusion of EV subpopulations with different sizes or densities.

Depending on the biofluid, we implement different protocols consisting of sequential separation methods with the aim to deplete non-EV particles (e.g., lipoprotein particles in blood plasma, THP in urine) and thus prepare EV with high specificity. Consequently, we study blood plasma EV of approximately 50–250 nm in size and 1.10–1.11 g/mL in density, and urinary EV of 1.10–1.11 g/mL in density. Although, the downstream analyses in this study are limited to particle and protein-based assays, we anticipate similar results for RNA-based assays, as previously reported [18].

Recent literature has demonstrated that storage conditions can influence EV recovery and function [37, 38]. To prevent bias, there was no difference in storage time of body fluids or (r)EV preparations per operator type. EV separations and characterizations were performed ad random and not per operator type.

A limitation of our study is the intralaboratory assessment that needs future validation by interlaboratory evaluation. However, based on the current data, we support the introduction of automated liquid handlers in EV separation protocols to facilitate EV biomarker discovery and clinical translation.

## Conclusions

In conclusion, automated liquid handling limits hands-on time and costs and ensures EV separation from body fluids with the highest reproducibility and specificity independent of the matrix used.

## Methods

### Sample collection and crude extract preparation

#### Blood plasma

Venous blood from breast cancer patients was collected using citrate blood collection tubes (455,322, Greiner Bio-one). Platelet-depleted plasma was prepared by two serial centrifugations at 2500 g for 15 min at room temperature. All blood samples were first characterized (complete blood count) using the hematology analyzer (XP-300, Sysmex). All blood samples were processed within 120 min after blood collection and platelet-depleted plasma was stored as 1 mL aliquots at −80 °C. Patient characteristics are summarized in Additional file 1: Fig. S1A. Crude extracts from total blood plasma were prepared using SEC columns with Sepharose CL-2B as previously described [10] (Fig. 1A). A SEC column was prepared by placing a nylon net with 20 μm pore size (NY2002500, Merck Millipore) on the bottom of a 10 mL syringe (3SYR-10ML, Romed), followed by stacking of 10 mL pre-washed Sepharose CL-2B (17,014,001, GE Healthcare). On top of one SEC column, 2 mL blood plasma was loaded followed by elution and collection of 6 sequential 1 mL eluate fractions. Following SEC, eluted fractions 4–5–6 were pooled and concentrated to 1 mL using a 10 kDa centrifugal filter (Amicon Ultra-2 mL, UFC201024, Merck Millipore) (referred to as the crude extract). The crude extracts were pooled and aliquots of 1 mL were stored at −80 °C [20].

#### Urine

Urine from healthy volunteers was collected and crude extracts were prepared as previously described [8]. Urine samples were centrifuged for 10 min at 1000 g and 4 °C. Cell-free urine supernatants were collected (leaving approximately 0.5 cm urine above the cell pellet). Cell-free urine samples (50 mL) were concentrated to 800 µL using a 10 kDa centrifugal filter device (Centricon Plus-70, UFC701008, Merck Millipore) (Fig. 1A) and stored at −80 °C.

All samples were collected in compliance with the Ethical Committee from Ghent University Hospital (approval EC/2014/0655) and relevant guidelines.

### EV separation by density gradient centrifugation

#### Operators and automated workstation

Manual density gradient preparation and fraction collection was performed by operators using a P1000 single channel pipette. The operators were divided in two groups based on their experience. Experienced operators were defined as having prepared more than 15 density gradients. Inexperienced operators had prepared one to five gradients maximum. All inexperienced operators were educated by the same instructor in the procedure by demonstration of the technique, received pipetting technique training, and were guided during the preparation and collection procedure.

Robot-assisted density gradient preparation and fraction collection was performed using the Biomek 4000 laboratory automation workstation (Beckman Coulter, A99749) with a custom-made script as previously described [10] (details are provided in the Additional file 1). The Biomek 4000 automation workstation has 12 deck positions and can pipet 1 µL up to 1000 µL by liquid-level sensing. To ensure sterility during the procedures, the automatic liquid handler was equipped with a positive-pressure HEPA enclosure. The workstation was used for the preparation of density gradients, sample loading, and collection of density gradient fractions.

#### Preparation of top-down and bottom-up OptiPrep density gradients

OptiPrep (60% (w/v) aqueous iodixanol solution, AXS-1,114,542, Axis-Shield) density gradients were prepared as previously described [9, 10]. Solutions of 5, 10, 20, and 40% iodixanol were made by mixing appropriate volumes of homogenization buffer (0.25 M sucrose (S0389, Sigma-Aldrich), 1 mM EDTA (1,084,180,100, Merck Millipore), 10 mM Tris (103,154 M, VWR) - HCL (44,921.K2, Alfa Aesar) (pH 7.4)) and iodixanol working solution. This working solution was prepared by combining a working solution buffer (0.25 M sucrose, 6 mM EDTA, 60 mM Tris-HCl (pH 7.4)) and a stock solution of OptiPrep. rEV were generated by transfection of HEK293T cells (CRL-11,268, ATCC) with gag-EGFP DNA followed by rEV separation from conditioned medium using density gradient centrifugation as described previously [29, 39].

A discontinuous top-down (TD) OptiPrep density gradient was made by layering 4 mL of 40%, 4 mL of 20%, 4 mL of 10% and 3.5 mL of 5% iodixanol solutions on top of each other in a 16.8 mL open top polyallomer tube (337,986, Beckman Coulter). 1 mL crude extract from blood plasma or phosphate-buffered saline (PBS, TMS-012-A, Merck Millipore) spiked with 1.85 × 10^10^ rEV (as quantified by fluorescent nanoparticles tracking analysis (fNTA) was overlaid on top of the gradient (Fig. 1B). (r)EV suspension was made by resuspending 800 µL crude extract from urine or PBS spiked with 1.85 × 10^10^ rEV (as quantified by fNTA) in 3.2 mL working solution, obtaining a 40% iodixanol suspension. A discontinuous bottom-up (BU) density gradient was prepared by overlaying 4 mL (r)EV suspension with 4 mL 20%, 4 mL 10% and 3.5 mL 5% iodixanol solutions, and 1 mL PBS (Fig. 1B).

TD and BU density gradients were centrifuged for 18 h at 100,000 g and 4 °C (SW 32.1 Ti rotor, Beckman Coulter).

The technique of manual preparing density gradients is described in Fig. 1C, Additional file 1: Box S1, and Additional file 2: Video S1.

For the automated preparation of density gradients, the single- and eight-channel P1000 pipetting tools, tip boxes (B01122, Beckman Coulter), pre-cooled iodixanol solutions reservoir, pre-cooled tube rack with the centrifuge tube(s), and sample rack were placed in one of the deck positions of the workstation (Fig. 1D). For density gradient preparation the eight-channel MP1000 tool was used (Additional file 3: Video S2), and for sample loading the single-channel P1000SL tool (Additional file 4: Video S3). The custom-made script allows the preparation of up to eight density gradients within one run.

#### Collection of density gradient fractions

After ultracentrifugation, density gradient fractions of 1 mL were collected from top to bottom manually or by the liquid handler.

The technique of manual fraction collection is described in Fig. 1C, Additional file 1: Box S1, and Additional file 5: Video S4.

The automated fraction collection requires the single-channel P1000 pipetting tool, tip box(es), pre-cooled tube rack with density gradient(s), and fraction rack(s) (Fig. 1D, Additional file 6: Video S5).

### EV recovery by ultracentrifugation or size-exclusion chromatography

To perform LC-MS/MS, ultracentrifugation was preferred as the most practical method to remove OptiPrep from individual EV-enriched density fractions from blood plasma and urine in a reproducible way, as previously described [10]. Density fractions 9 and 10 were separately transferred to centrifuge tubes. 14 mL of pre-cooled PBS was added to each sample and the solution was mixed in the tube by pipetting up and down. The tubes were centrifuged for 3 h at 100,000 g and 4 °C (SW 32.1 Ti rotor, Beckman Coulter). After ultracentrifugation, the supernatant was discarded leaving 50 µL at the bottom of the tube. The pellet was diluted to 100 µL with pre-cooled PBS. To prevent loss of EV sticking to the bottom of the tube, the EV pellet was directly lysed in the tube. Lysates were prepared by mixing samples with SDT-lysis buffer (2% SDS (436143-25G, Sigma-Aldrich), 500 mM Tris (103,154 M, VWR) - HCL (44,921.K2, Alfa Aesar) (pH 7.6), 0.5 M dithiothreitol (39759.02, Serva)) at a 4:1 sample to buffer ratio. The pellet was pipetted up and down and the bottom of the tube was rinsed with SDT-lysis buffer. The lysates were collected and incubated at 95 °C for 5 min. Lysates were stored at − 80 °C until processing for LC-MS/MS.

To perform transmission electron microscopy (TEM), EV were separated from pooled density fractions 9–10 obtained from blood plasma and urine samples by including a second SEC, following previously mentioned protocol unless stated otherwise. From this second SEC, eluted size-based fractions 4-5-6-7 were pooled and concentrated to 100 µL using a 10 kDa centrifugal filter (Amicon Ultra-2 mL, UFC201024, Merck Millipore) and stored at −80 °C.

### Density measurement

The density of the density-gradient fractions was calculated using a standard curve of the absorbance values at 340 nm (SpectraMax Paradigm, Molecular Devices) of 1:1 aqueous dilution of 5, 10, 20 and 40% iodixanol solutions. This standard curve was used to determine the density of fractions collected from a control gradient overlaid with 1 mL of PBS.

### Interface mixing

To determine interface mixing, the image of colored test density gradients prepared by an inexperienced, experienced, and automated operator was analyzed using ImageJ software version 1.53. Each 10% iodixanol layer was individually circumscribed with the rectangular region of interest (ROI) selection. Colors were converted to binary. Profile plots of the ROIs were generated. Area measurement of the peaks (corresponding with the spilling of the 5% and 20% iodixanol solution in the 10% layer) was performed with the wand tool.

### Fluorescent nanoparticle tracking analysis

Fractions of rEV spiked density gradients were analyzed by fluorescent nanoparticle tracking analysis (fNTA) using a NanoSight LM10-HS microscope (Malvern Instruments Ltd) equipped with a 488 nm laser, an additional 500 nm longpass filter and an automatic syringe pump system (infusion speed: 20) (Fig. 1E). For each analysis, three videos of 60 s were recorded and analyzed with camera level 16 and detection threshold 3. Temperature was monitored during recording. Recorded videos were analyzed with the NTA Software version 3.3. For optimal measurements, samples were diluted with PBS until particle concentration was within the concentration range for the NTA Software (3 × 10^8^-10^9^ particles/mL). For recovery calculations the number of fluorescent particles was measured before spiking.

### Anti-p24 ELISA

Gag-EGFP protein concentrations in fractions of rEV spiked density gradients were determined with the anti-p24 ELISA kit Innotest HIV antigen mAb (80,563, Fujirebio) (Fig. 1E) according to the manufacturer’s instructions. For recovery calculations a rEV standard curve, from the same batch as used for spiking, was included ranging from 1 × 10^6^-10^7^ fluorescent particles as previously measured with fNTA.

### Western blot

Protein concentrations of rEV were measured, after lysis with 0.2% SDS (436143-25G, Sigma-Aldrich), with the Qubit Protein Assay (ThermoFisher) and Qubit fluorometer 3.0 following manufacturer’s instructions. For protein analysis, samples were dissolved in reducing sample buffer (0.5 M Tris-HCl [pH 6.8], 40% glycerol, 9.2% SDS, 3% 2-mercaptoethanol, 0.005% bromophenol blue) and boiled at 95 °C for 5 min. Proteins were separated by SDS-PAGE and transferred to nitrocellulose membranes (Bio-Rad). Membranes were blocked for 30 min in blocking buffer (5% non-fat milk in PBS with 0.5% Tween-20) and incubated overnight at 4 °C with primary antibodies (mouse monoclonal anti-ALIX (1:1000, #2171); rabbit monoclonal anti-CD9 clone D3H4P (1:1000, #13403S) (Cell Signaling Technology); and mouse monoclonal anti-flotillin-1 (1:1000, #610,820) (BD Biosciences)). Secondary antibodies (sheep anti-mouse horseradish peroxidase-linked antibody (1:3000, #NA931V) and donkey anti-rabbit horseradish peroxidase-linked antibody (1:4000, #NA934V) (GE Healthcare Life Sciences)) were added for 60 min at room temperature after extensive washing with blocking buffer. After final washing, chemiluminescence substrate (WesternBright Sirius, Advansta) was added and imaging was performed using Proxima 2850 Imager (IsoGen Life Sciences).

### Protein measurements

Protein concentrations of the lysed EV preparations obtained from blood plasma and urine samples were measured using the fluorometric Qubit Protein Assay (ThermoFisher) and the Qubit Fluorometer 3.0 (ThermoFisher) according to the manufacturer’s instructions.

### LC-MS/MS

EV preparations obtained from blood plasma and urine samples were processed for LC-MS/MS by filter-aided sample preparation (FASP) [40] (Fig. 1E). After thawing and clarification by centrifugation (16,000 g for 5 min), lysates were mixed with 300 µL UA (8 M urea (U5128, Sigma-Aldrich), 0.1 M Tris-HCl (pH 8.5)) in a Microcon-10 kDa centrifugal filter device (MRCPRT010, Merck Millipore). Filters were centrifuged twice (14,000 g for 40 min at 20 °C) with the addition of 200 µL UA in between. Proteins were alkylated by addition of 100 µL IAA solution (0.05 M iodoacetamide (I1149, Sigma-Aldrich) in UA buffer) and incubated for 30 min at room temperature, followed by centrifugation. Samples were treated twice by addition of 100 µL UA and centrifugation. Subsequently, samples were twice treated by addition of 100 µL DB buffer (1 M urea, 0.1 M Tris-HCl (pH 8.5) and centrifugation. Filter units were transferred to new collection tubes and proteins were resuspended in 40 µL DB with Trypsin/Lys-C mix (V5073, Promega) for overnight proteolytic digestion at 37 °C. Digests were collected by addition of 100 µL DB and centrifugation for 15 min at 14,000 g. This step was repeated once. Collected peptides were acidified with 1% trifluoroacetic acid to a pH of 2–3, followed by desalting with Peptide Cleanup C18 Spin Tubes (5188 − 2750, Aligent). Desalted peptides were vacuum dried, dissolved in 0.1% formic acid and analyzed by LC-MS/MS. Equal amounts of peptides of each sample were loaded on a nanoflow HPLC system (Easy- nLC1000, Thermo Fisher Scientific) coupled to a Q Exactive HF Hybrid Quadrupole-Orbitrap Mass Spectrometer (Thermo Fisher Scientific) equipped with a nano-electrospray ionization source. The mobile phase consisted of 0.1% formic acid (solvent A) and acetonitrile/water (95:5 (v/v)) with 0.1% formic acid (solvent B). The peptides were separated with a 40 min gradient from 8 to 35% of solvent B. Before the end of the run, the percentage of solvent B was raised to 100% in 2 min and kept there for 8 min. Full MS scan over the mass-to-charge (m/z) range of 300–1750 with a resolution of 120,000, followed by data dependent acquisition with an isolation window of 2.0 m/z and a dynamic exclusion time of 30 s was performed. The top 12 ions were fragmented by higher energy collisional dissociation (HCD) with a normalized collision energy of 27% and scanned over the m/z range of 200–2000 with a resolution of 15,000. After the MS2 scan for each of the top 12 ions had been obtained, a new full mass spectrum scan was acquired, and the process repeated until the end of the 50 min run. Three repeated runs per sample were performed. Tandem mass spectra were searched using the MaxQuant software (version 1.6.10.43) against a database containing reviewed sequences of homo sapiens of UniProtKB release 2019_11. Peptide-spectrum-match- and protein-level false discovery rates were set at 0.01. Carbamidomethyl (C), as a fixed modification, and oxidation (M) and acetylation of the protein N-terminus as dynamic modifications were included. A maximum of two missed cleavages was allowed. The LC-MS profiles were aligned, and the identifications were transferred to non-sequenced or non-identified MS features in other LC-MS runs (matching between runs). The protein was determined as detected in the sample if its identification had been derived from at least two unique peptide identifications. Filtering for contaminating proteins, reverse identification and identification by site was used. Label-free quantification (LFQ) was performed using the MaxLFQ algorithm integrated in the MaxQuant software.

### Proteomic data analysis

Identified proteins were analyzed and visualized using Perseus software version 1.6.15.0 [41]. Proteins showing valid values in at least 70% of at least one group were selected. Reverse database hits and potential contaminant proteins were removed. Missing values were imputed from the observed normal distribution of intensities. LFQ intensities were normalized using the Width adjustment method in Perseus. For selected analyses, the normalized LFQ intensities were log2 transformed. Coefficient of variation (CV) analysis was based on the 100 highest quantified proteins within each sample type. Principal component analysis (PCA) was performed using the Perseus software. Unsupervised hierarchical clustering heat maps, using 1-Pearson correlation, were generated using the Morpheus tool. Analysis of similarities (anosim) was performed using Past3 software [42]. The Vesiclepedia database was explored to identify the 100 most common EV-associated proteins [43]. Quantitative expression profile based functional enrichment analysis was performed FunRich software version 3.1.3 [44].

### Transmission electron microscopy

EV preparations obtained from blood plasma and urine were qualitatively analyzed with transmission electron microscopy (TEM) (Fig. 1E). Samples were deposited on a formvar coated grids stabilized with evaporated carbon film and glow discharged before sample application (AGS162-3 H, Agar Scientific). Neutral uranyl acetate (2% in AD) (21447-25, Polysciences) was used for staining after which grids were coated with 2% methyl cellulose (M7027, Sigma-Aldrich) / uranyl acetate (0,4%) solution. These grids were examined using a Tecnai G2 Spirit transmission electron microscope (Thermo Fisher Scientific FEI) operated at 100 kV and images were captured with a Quemesa charge-coupled device camera (Olympus Soft Imaging Solutions).

Information on density measurement, interface mixing, and characterization methods **(**Fig. 1E**)** of rEV (fNTA and anti-p24 ELISA) and EV (mass-spectrometry based proteomics and TEM) is provided in the Additional file 1.

### Statistical analysis

Data analysis and visualization was performed using GraphPad Prism version 8 (GraphPad Software). Data are expressed as median with interquartile range (IQR). Correlations were calculated using the Pearson product-moment (r). Differences of mean ranks were evaluated by Mann Whitney U test and differences of variance by F-test of equality of variances. P-values smaller than 0.05 were considered statistically significant.

## Supplementary Information

**Additional file 1: Figure S1. **A) Baseline clinical characteristics of the breast cancer patients included in the study. B) Correlation analysis between rEV levels based on fluorescent NTA and anti-p24 ELISA after bottom-upand top-downdensity-based separation. C) Western blot analysis of density fractions obtained after manual TD density gradient centrifugation of rEV-spiked cell culture supernatans from HCT116. Concentration and size distribution in 1:8 dilution of density fraction 9under fluorescence NTA mode after D) BU experienced and E) TD automated density-based rEV separation. **Figure S2. **LC-MS/MS data from EV-enriched density fractions 9 and 10 obtained from blood plasmaand urineafter density-based separation byexperienced operatorsand automatedliquid handlingare compared by A) venn diagram, B) anosim analysis, and C) hierarchical clustering. Source data are provided as Additional file 8: Table S2.** Figure S3. **A) Time to prepare and fractionate 3 density gradients by manualand automatedliquid handling. B) Cost distribution of manual and automated density gradient centrifugation. Yearly operating cost is based on moderateor high performance of density gradient centrifugation. C) Cost per gradient by manual or automated density gradient preparation and fractionation in function of the number of gradients. Source data are provided as Additional file 9: Table S3. **Box S1**. Step-by-step procedure. **Box S2.** Evaluation of the technique.

**Additional file 2: Video S1. **Manual density gradient preparation.

**Additional file 3: Video S2. **Automated density gradient preparation.

**Additional file 4: Video S3. **Automated sample loading.

**Additional file 5: Video S4. **Manual fraction collection.

**Additional file 6: Video S5. **Automated fraction collection.

**Additional file 7: Table S1. **rEV data.

**Additional file 8: Table S2. **MS/MS data.

**Additional file 9: Table S3. **Cost calculations.

## Data Availability

The mass spectrometry proteomics dataset supporting the conclusions of this article is available in the ProteomeXchange Consortium via the PRIDE partner repository, with the dataset identifier PXD032394 via http://proteomecentral.proteomexchange.org/cgi/GetDataset. We have submitted all relevant experimental parameters to the EV-TRACK knowledgebase (EV-TRACK ID: EV210215 via https://evtrack.org/) [5]. The datasets supporting the conclusions of this article are included within the Supplemental Tables.

## Abbreviations

EV: Extracellular vesicles
rEV: Recombinant extracellular vesicles
SEC: Size‐exclusion chromatography
THP: Tamm-Horsfall protein
TD: Top-down
PBS: Phosphate-buffered saline
fNTA: Fluorescent nanoparticle tracking analysis
BU: Bottom-up
ROI: Region of interest
FASP: Filter-aided sample preparation
LFQ: Label-free quantification
PCA: Principal component analysis
Anosim: Analysis of similarities
TEM: Transmission electron microscopy
Man.: Manual
Inexp.: Inexperienced
Exp.: Experienced
Auto.: Automated
CV: Coefficient of variation
GOCC: Gene ontology cellular component
