# Supplementary material for: Integrating automated liquid handling in the separation workflow of extracellular vesicles enhances specificity and reproducibility
Source: J Nanobiotechnology. 2023 May 19;21:157. doi: 10.1186/s12951-023-01917-z (PMC10197845; doi:10.1186/s12951-023-01917-z)
Supplement: Supplementary file 1 — Additional file 1: Figure S1. A) Baseline clinical characteristics of the breast cancer patients included in the study. B) Correlation analysis between rEV levels based on fluorescent NTA and anti-p24 ELISA after bottom-upand top-downdensity-based separation. C) Western blot analysis of density fractions obtained after manual TD density gradient centrifugation of rEV-spiked cell culture supernatans from HCT116. Concentration and size distribution in 1:8 dilution of density fraction 9under fluorescence NTA mode after D) BU experienced and E) TD automated density-based rEV separation. Figure S2. LC-MS/MS data from EV-enriched density fractions 9 and 10 obtained from blood plasmaand urineafter density-based separation byexperienced operatorsand automatedliquid handlingare compared by A) venn diagram, B) anosim analysis, and C) hierarchical clustering. Source data are provided as Additional file 8: Table S2. Figure S3. A) Time to prepare and fractionate 3 density gradients by manualand automatedliquid handling. B) Cost distribution of manual and automated density gradient centrifugation. Yearly operating cost is based on moderateor high performance of density gradient centrifugation. C) Cost per gradient by manual or automated density gradient preparation and fractionation in function of the number of gradients. Source data are provided as Additional file 9: Table S3. Box S1. Step-by-step procedure. Box S2. Evaluation of the technique. [file 12951_2023_1917_MOESM1_ESM.pdf]

SUPPLEMENTAL METHODS

Settings for density gradient preparation and fractionation by automated liquid handling

The pipetting variables are provided in the table below.

	PREPARATION		FRACTIONATION	
	**Make_Gradient**	**Add_Sample**	**Make_Fraction**	**Waste_Fraction**
**Aspirate_Speed**	150 µL/s	50 µL/s	150 µL/s	150 µL/s
**AspirateFromLiquid**	-2 mm	-2 mm	-2 mm	-2 mm
**Dispense_Speed**	100 µL/s	100 µL/s	75 µL/s	100 µL/s
**DispenseFromLiquid**	5 mm	0 mm	2 mm	2 mm
**Down_Speed**	50%	50%	50%	50%

Description of the variables:
- Aspirate_Speed: speed of aspirating liquid (µL/s)
- AspirateFromLiquid: distance from the liquid (mm) at which aspiration occurs
- Dispense_Speed: speed of dispensing liquid (µL/s)
- DispenseFromLiquid: distance from the liquid (mm) at which dispensing occurs
- Down_Speed: speed to move within the well (% of maximum speed of the pod)

Importantly, ‘liquid level when aspirating or dispensing liquid’ is selected during all procedures (tip follows liquid level during pipetting operations). Liquid level is determined using specially designed liquid level sensing tips that detect a shift in the capacitance of the probe. The tip moves to a specified height within the well and then slowly moves down into the well. When the tip contacts liquid, there is a large change in capacitance detected. The liquid level is sensed by determining the height at which this change in capacitance occurs. The following settings are entered for liquid level sensing: ‘move to 0 mm from the top before sensing’ and ‘sense at 25% speed’.

Additionally, during fractionation, ‘aspirate a trailing air gap leaving the liquid’ is enabled to help prevent drips.

SUPPLEMENTAL FIGURES

A

**Number of patients**	14
**Mean age (range)**	52 (35-65)
**Type**	
IDC	6
ILC	0
NST	8
**Tumor grade**	
1	0
2	3
3	10
Unknown	1
**Immunohistochemistry**	
ER positive	5
PR positive	5
HER2neu amplification	4
**Treatment**	
4x EC dd q2w + 12x taxol q1w	12
12x carbo-taxol q1w + 4x EC dd q2w	2
Radiotherapy	14

IDC: invasive ductal carcinoma, ILC: invasive lobular carcinoma, NST: no special type, ER: estrogen receptor, PR: progesterone receptor, EC dd: epirubicin-cyclophosphamide dose dense

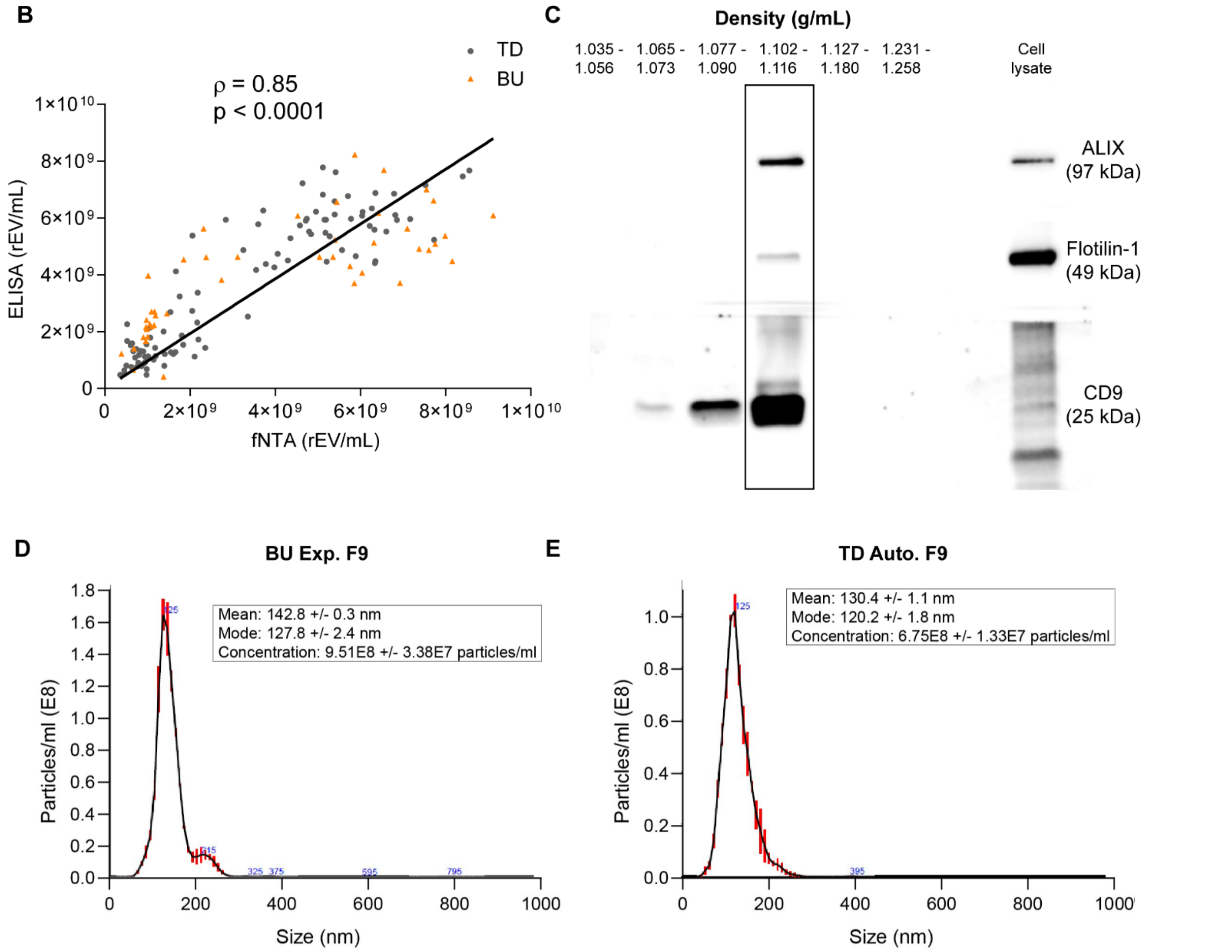

Supplemental Figure 1. A) Baseline clinical characteristics of the breast cancer patients included in the study. **B)** Correlation analysis between rEV levels based on fluorescent NTA and anti-p24 ELISA after bottom-up (BU) and top-down (TD) density-based separation (Pearson's r = 0.85, p < 0.0001). **C)** Western blot analysis of density fractions obtained after manual TD density gradient centrifugation of rEV-spiked cell culture supernatans from HCT116 (2 x 10^{10} rEV/mL). Concentration and size distribution in 1:8 dilution of density fraction 9 (F9) under fluorescence NTA mode after **D)** BU experienced and **E)** TD automated density-based rEV separation.

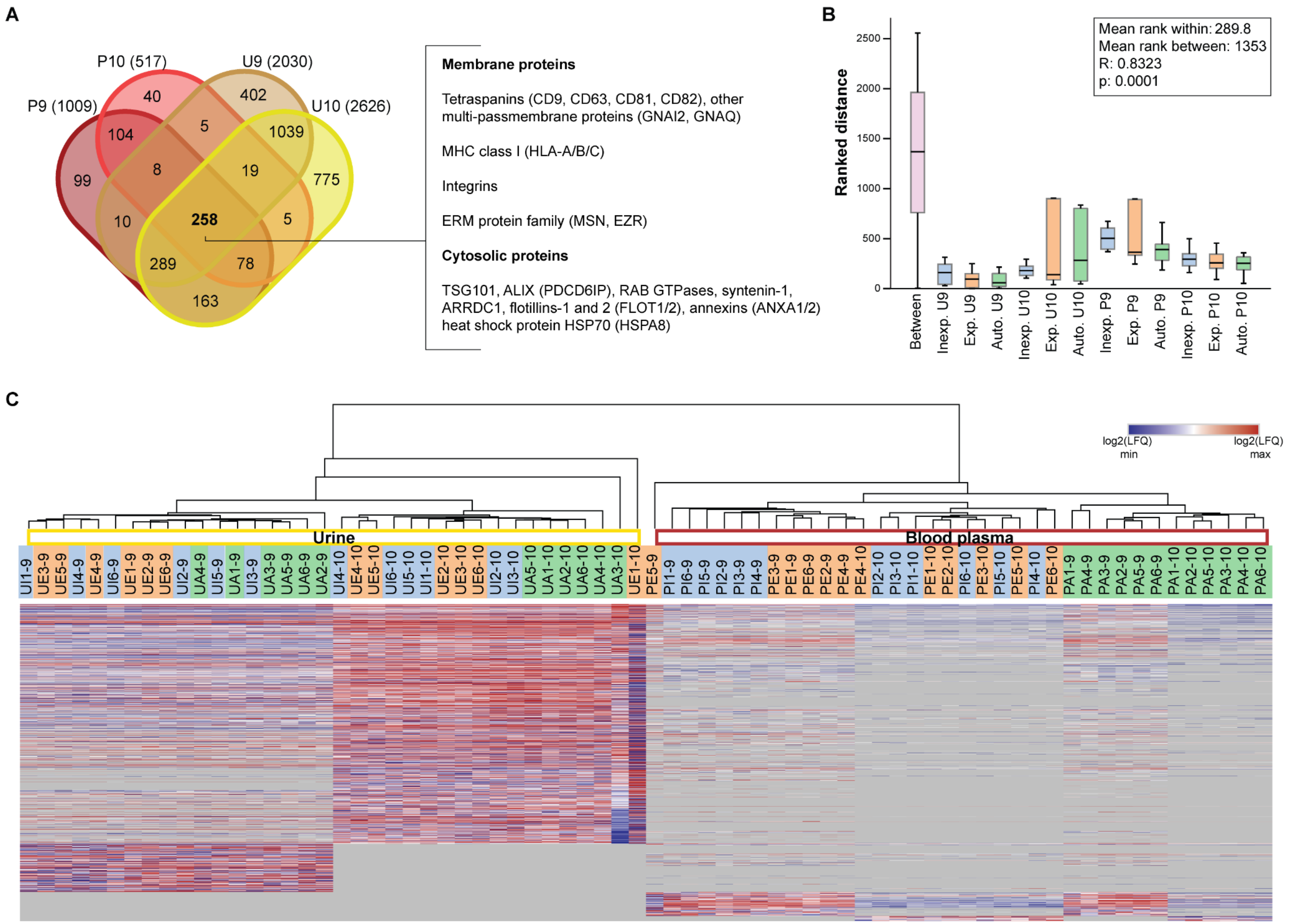

Supplemental Figure 2. LC-MS/MS data from EV-enriched density fractions 9 and 10 obtained from blood plasma (P9, P10) and urine (U9, U10) after density-based separation by (in)experienced operators (inexp., exp.) and automated (auto.) liquid handling (n = 6) are compared by **A)** venn diagram, **B)** anosim analysis, and **C)** hierarchical clustering. Source data are provided as Supplemental Table 2.

A **Time per 3 gradients (in min)**

	Man.	Auto.	
		Hands-on	Liquid handler
Preparation	60	2	25
Fractionation	45	3	24
**Total**	**105**	**54**	

B

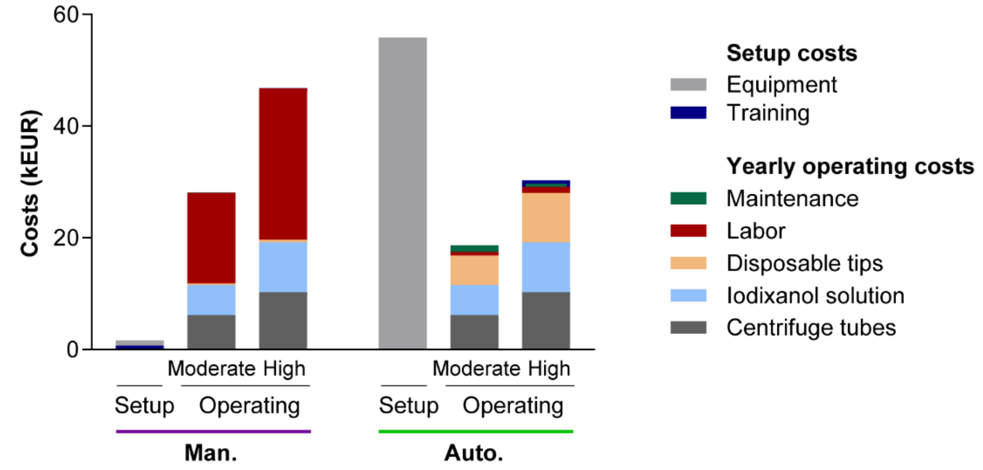

C

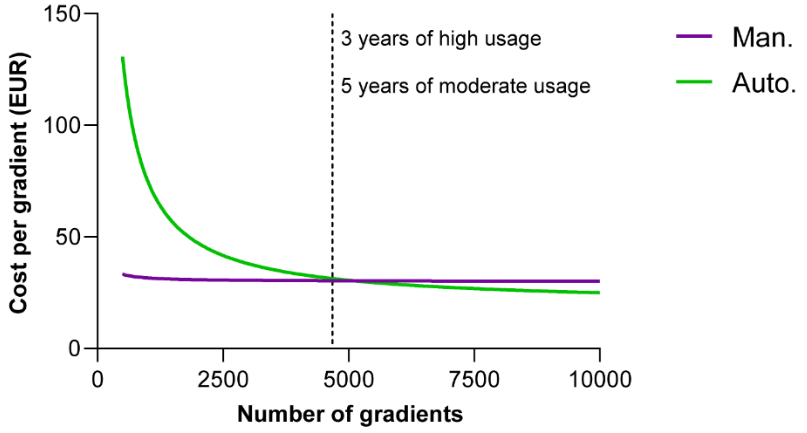

Supplemental Figure 3. A) Time to prepare and fractionate 3 density gradients by manual (man.) and automated (auto.) liquid handling. **B)** Cost distribution of manual and automated density gradient centrifugation. Yearly operating cost is based on moderate (18 gradients per week) or high performance of density gradient centrifugation (30 gradients per week). **C)** Cost per gradient by manual or automated density gradient preparation and fractionation in function of the number of gradients (cost per gradient = setup cost/number of gradients + operating cost per gradient). Source data are provided as Supplemental Table 3.

SUPPLEMENTAL BOXES

Supplemental Box 1: Step-by-step procedure

Preparation of density gradients (**Supplemental Video 1**):

1. Make use of a recently calibrated and lubricated P1000 pipette.
2. Carefully transfer 40% (wt/vol) iodixanol solution to the bottom of the centrifugation tube, holding the tube vertically.
3. Tilt the tube slowly to 70° and transfer 4 x 1 mL of the 20% iodixanol solution carefully, dropwise to the surface of the liquid (close to the opening of the tube) (**Fig. 1C**).
4. Next, transfer 4 x 1 mL of 10%, 4 x 875 µL of the 5% iodixanol solution, and 1 mL PBS or sample in similar manner.

Fractionation of density gradients (**Supplemental Video 4**):

1. Hold the centrifugation tube upright.
2. Carefully collect fractions by slowly pipetting 1 mL from the central bottom of the concave meniscus at the liquid surface using a P1000 pipette (**Fig. 1C**).

Supplemental Box 2: Evaluation of the technique

Colored test gradients:

The manual preparation and fraction collection of density gradients can be easily assessed by adding 50 µL trypan blue solution to the 20% and 5% (wt/vol) iodixanol solutions. The different layers should be sharply defined from one another in the prepared gradient with maximum 20% interface mixing per layer. During fraction collection, no liquid from the underlying fraction should be pipetted up (visual check by color).

(r)EV-spiked PBS gradients:

Spike a pre-defined number (minimum 1 x 10^{10}) of rEV or cell-derived EV per gradient. Both top-down and bottom-up approaches can be practiced. The competence of the researcher in the performance of the technique can be evaluated by NTA measurement of the different 1 mL density fractions. A (r)EV recovery of at least 30% in fraction 9 and 10 (density 1.096 - 1.113 g/mL) is expected with minimal spilling to the neighboring fractions. In case of rEV, anti-p24 ELISA can further validate these results.
